# Bidara Upas Leaf Extract (*Merremia mammosa*) as a Therapy to Enhance Pulmonary Macrophage Cells in Mice Infected With *Mycobacterium tuberculosis*

**DOI:** 10.1155/tswj/7252815

**Published:** 2026-03-14

**Authors:** Khambali, Rachmaniyah, Fitri Rokhmalia, Rosidi Roslan

**Affiliations:** ^1^ Department of Environmental Health, Health Polytechnic Ministry of Health Surabaya, Surabaya, Indonesia; ^2^ The Surabaya Health Quarantine Agency, Surabaya, Indonesia

**Keywords:** drug resistance, lung macrophages, *Merremia mammosa*, *Mycobacterium tuberculosis*, tuberculosis

## Abstract

Tuberculosis (TB) is a serious infection caused by *Mycobacterium tuberculosis*, primarily affecting the lungs and spreading through the air. Each year, approximately 10 million new TB cases are reported worldwide, with Indonesia ranking third in the highest number of TB cases. TB treatment faces challenges due to drug resistance, such as MDR‐TB and XDR‐TB, which drives research into alternative therapies, one of which is the extract of *Merremia mammosa*, known for its antibacterial and immunomodulatory potential. This study is aimed at evaluating the potential of *Merremia mammosa* extract in enhancing immune responses, particularly through lung macrophages, in mice infected with *M. tuberculosis*. The research was conducted at the Integrated Laboratory of the Poltekkes Kemenkes Surabaya, from March to December 2024. Mice were divided into treatment and control groups, receiving extracts at varying doses after TB bacteria injection. The results indicated that *Merremia mammosa* extract could reduce lung cell necrosis, with the treatment groups showing lower levels of necrosis compared to the positive control group. The P13 and P12 treatment groups showed the best results, with minimal to no necrosis, indicating the potential therapeutic effects of the extract in improving lung tissue. Appropriate dose adjustments are necessary for optimal lung protection.

## 1. Introduction

Tuberculosis (TB) remains a significant global health issue. This disease is caused by the bacterium *Mycobacterium tuberculosis*, which primarily infects the lungs, although it can also affect other organs. TB is a contagious disease that spreads through the air when an infected individual coughs or sneezes, releasing bacterial particles into the surrounding air [1]. According to the World Health Organization (WHO), approximately 10 million new TB cases are reported worldwide each year, with a death toll of around 1.5 million people in 2021. In Indonesia, TB remains one of the major health challenges, with the country ranking third after India and China in terms of the highest number of TB cases [2].

The current treatment for TB involves a combination of antibiotics over a period of 6–9 months, with primary drugs such as rifampicin, isoniazid, pyrazinamide, and ethambutol. However, this therapy faces several challenges, including patient adherence to the long regimen, serious side effects of the medications, and the emergence of drug‐resistant cases such as multidrug‐resistant tuberculosis (MDR‐TB) and extensively drug‐resistant tuberculosis (XDR‐TB) [3, 4]. MDR‐TB is a form of TB that is resistant to two main drugs, namely, rifampicin and isoniazid, which are the first‐line drugs used in TB treatment, thereby requiring the use of second‐line drugs that are more expensive, more toxic, and involve longer treatment duration. XDR‐TB is a more severe form of MDR‐TB that is also resistant to several second‐line drugs, making treatment even more difficult and limited to highly expensive therapeutic options. Drug resistance in TB further complicates the treatment and control of the disease, as patients with MDR‐TB and XDR‐TB require longer and more complex treatment, often involving medications with more severe side effects.

TB is an infectious disease that remains a global health issue, with high numbers of cases and deaths in various countries. According to a report from the WHO, it was estimated that around 1.7 billion people were infected with TB in 2019 [5]. Each year, there are approximately 10 million new TB cases, with 1.2 million deaths among people who are not infected with HIV and 251,000 deaths among people with HIV. TB is also one of the main factors exacerbating poverty and hindering social development [6]. In Vietnam, the WHO estimated that there were 174,000 new TB cases and 13,200 TB‐related deaths in 2018. Currently, a serious issue is the increasing number of drug‐resistant TB cases (MDR‐TB). According to the 2019 report from the National TB Program (NTP), the prevalence of MDR‐TB among new patients reached 3.6%, while among patients receiving retreatment, it was as high as 17% [7].

As drug resistance increases, the need for safer and more effective alternative and complementary therapies in the treatment of TB has become more urgent. In recent decades, several studies have focused on the use of traditional medicinal plants that have potential as natural antimicrobial agents and immunomodulators [8]. The use of medicinal plants has the potential not only as an adjunct therapy but also as a source of active compounds that can address drug resistance with a lower risk of side effects compared to chemical drugs. One plant that has attracted attention as a complementary therapy for TB is *Merremia mammosa*, which has long been used in traditional medicine in Indonesia to treat various infections, including respiratory tract infections [9].

*Merremia mammosa* is known to contain various bioactive compounds, such as flavonoids, alkaloids, saponins, and tannins, which exhibit a range of pharmacological activities, including antibacterial, anti‐inflammatory, antioxidant, and immunomodulatory effects [10]. These compounds are believed to help inhibit the growth of *M. tuberculosis* and enhance the body′s immune response in combating the infection. A study conducted showed that *Merremia mammosa* extract could enhance the phagocytic activity of macrophages in mice infected with *M. tuberculosis*, indicating a significant immunomodulatory effect. Additionally, the flavonoids in *Merremia mammosa* are also known to possess strong antioxidant activity, which can protect cells from oxidative damage caused by infection [11].

Macrophages are the primary immune cells that play a crucial role in the innate immune response to TB infection. When *M. tuberculosis* enters the body, macrophages attempt to eliminate the bacteria through the process of phagocytosis. However, *M. tuberculosis* has the ability to survive within macrophages by inhibiting the phagolysosome process, which allows the bacteria to proliferate inside the cell and worsen the infection [12]. Therefore, enhancing macrophage activity in response to TB infection is an important approach in TB therapy. A study showed that administration of *Merremia mammosa* extract in a TB‐infected animal model could enhance the phagocytic activity of macrophages, potentially improving the body′s immune response to *M. tuberculosis* infection.

The flavonoids found in *Merremia mammosa* are known to enhance the activity of antioxidant enzymes such as superoxide dismutase (SOD), catalase, and glutathione peroxidase, which are important enzymes in the body. This antioxidant activity can help reduce oxidative stress caused by TB infection, which often leads to damage to immune cells, including macrophages. By boosting antioxidant activity, *Merremia mammosa* can support the maintenance of normal macrophage function, allowing these cells to be more effective in eliminating *M. tuberculosis* from the body. Furthermore, flavonoids are also known to inhibit the production of proinflammatory cytokines such as TNF‐*α*, IL‐1*β*, and IL‐6, which contribute to the chronic inflammation seen in TB.

In addition to flavonoids, the alkaloids contained in *Merremia mammosa* also possess strong antibacterial potential, which can help inhibit the growth of *M. tuberculosis*. Alkaloids work by damaging the bacterial cell wall structure, leading to leakage and bacterial death. Research shows that alkaloids can work synergistically with standard antibiotics, enhancing the effectiveness of TB treatment, potentially reducing the required antibiotic dosage, and decreasing the risk of drug resistance. An in vitro study also demonstrated that *Merremia mammosa* extract has significant bactericidal activity against *M. tuberculosis*, further strengthening the potential of this plant as a complementary therapy in TB treatment [13].

*Merremia mammosa* has long been used in traditional medicine in Indonesia, particularly by communities in Java and Sumatra, to treat various infectious and inflammatory diseases. This plant is typically prepared in the form of a decoction or extract that is consumed orally or used topically to treat open wounds. In traditional medicine, *Merremia mammosa* is used to address health issues such as respiratory infections, digestive disorders, and burns. Several ethnobotanical studies indicate that *Merremia mammosa* has a good reputation as a safe and effective medicinal plant, with few reports of side effects or toxicity at therapeutic doses [14].

However, despite the long history of *Merremia mammosa* in traditional medicine, scientific research on its effectiveness and safety in the treatment of TB remains limited. Therefore, this study is aimed at assessing the potential of *Merremia mammosa* extract as a pharmacological agent and immunomodulator in the treatment of TB, focusing on its effects on the activity of pulmonary macrophages in mice infected with *M. tuberculosis*. This study will also evaluate different doses of *Merremia mammosa* extract to determine the optimal dose that provides the best immunomodulatory effect without causing toxicity. Based on this, the study is aimed at investigating the application of *Merremia mammosa* extract as a therapy to improve lung health quality in patients with pulmonary TB. The research will examine the therapeutic effects of *Merremia mammosa* on the overall condition of the body, particularly in enhancing the activity of pulmonary macrophages to eliminate *M. tuberculosis*, strengthen immunity, and reduce inflammation caused by the infection.

## 2. Method

This study used five treatment groups, namely, mice infected with *M. tuberculosis*, whose lung organs were taken, sectioned, embedded in paraffin, and stained with hematoxylin–eosin (H&E) before being observed under a microscope via intraperitoneal injection. Following infection, *M. tuberculosis* colonizes the lungs of mice within 14–21 days, with pulmonary pathology and measurable immune responses typically emerging during this period. Therefore, in this study, a 21‐day postinfection period was used to allow disease establishment before treatment began. The presence of *M. tuberculosis* infection was confirmed by histopathological examination of lung tissue stained with H&E, which revealed inflammatory infiltrates, alveolar damage, and necrosis. These findings are consistent with murine TB models that demonstrate pulmonary pathology within 2–4 weeks postinfection [15, 16].

The tubers (*Merremia mammosa*) were collected, cleaned, and prepared for extraction. The extraction was performed using 70% ethanol as a solvent through maceration and circulation methods. After the extraction process was completed, the filtrate was concentrated using a rotary evaporator until a thick extract was obtained. The extract was then prepared at the desired concentrations (50, 100, and 150 ppm) according to the doses administered to mice in the treatment groups.

Similarly, 35 mice were randomly assigned to five groups, with each group containing seven mice. Groups 1–5 are observation groups, which are further divided into preobservation (O1–O5) and postobservation (O6–O10). Groups P0–P4 are treatment groups, namely:

a. P1 = Treatment 1 (injected with *M. tuberculosis* and given *Merremia mammosa* extract at 50 ppm);
b. P2 = Treatment 2 (injected with *M. tuberculosis* and given *Merremia mammosa* extract at 100 ppm);
c. P3 = Treatment 3 (injected with *M. tuberculosis* and given *Merremia mammosa* extract at 150 ppm);
d. K− = control group, only given *M. tuberculosis* bacteria;
e. K+ = control/comparative group that was given *M. tuberculosis* and rifampicin (P1, P2, and P3 each received a 2 mL/200 g body weight dose of extract orally).

The assessment of cell necrosis in this study used the Manja Roenigk scoring system, which provides a description of cell damage quality based on a scale from 1 to 4. The Manja Roenigk score is based on the following criteria:

I. Score 1 indicates a normal number of cells, with no damage or degeneration;
II. Score 2 indicates parenchymal degeneration, which is a structural change in the cells without noticeable cell death;
III. Score 3 indicates hydropic degeneration, where the cells swell due to fluid accumulation;
IV. Score 4 indicates necrosis, or cell death resulting from severe damage to the cell structure, which can be caused by infection, inflammation, or other stress factors.

In this study, ethical approval was obtained from the Department of Environmental Health, Health Polytechnic Ministry of Health Surabaya, Surabaya, Indonesia, No. EA/2126/KEPK‐Poltekkes_Sby/V/2024. It was deemed ethically appropriate in accordance with the seven WHO 2011 standards, which are (1) social value, (2) scientific value, (3) fair distribution of burdens and benefits, (4) risk, (5) coercion/exploitation, (6) confidentiality and privacy, and (7) informed consent, referring to the CIOMS Guidelines 2016. This is demonstrated by the fulfillment of each standard′s indicators.

## 3. Results and Discussion

TB remains a major global health challenge, despite progress in treatment. In 2022, the WHO reported over 10 million new cases of TB, with high mortality rates, particularly in developing countries. Drug resistance is a major issue in TB treatment, where *M. tuberculosis* resistant to standard medications further exacerbates the situation. Therefore, the development of new, more effective, and safer therapies is a priority in TB treatment [17]. One promising alternative is the use of traditional medicinal plants, such as *Merremia mammosa*, which is known to contain various bioactive compounds with antibacterial and anti‐inflammatory activities. Previous research has shown that *Merremia mammosa* extract can function as an immunomodulatory agent that enhances the activity of macrophages, which play an important role in combating *M. tuberculosis*. As part of the immune system, macrophages have the ability to ingest and destroy pathogenic bacteria, including *M. tuberculosis*. Therefore, increasing the number and function of macrophages could accelerate the recovery of TB patients [18, 19].

This study is highly relevant because the use of *Merremia mammosa* as a complementary therapy for TB has yet to be explored in depth. As a traditional medicinal plant with antibacterial potential, *Merremia mammosa* can be used as an adjunct therapy to reduce dependence on more expensive pharmaceutical drugs with potential side effects. This is also important for improving the effectiveness of TB treatment, especially with the growing number of drug‐resistant cases [20, 21].

### 3.1. Necrosis in Mice Infected With *M. tuberculosis*

Cell necrosis is the process of cell death resulting from irreversible cell damage, typically caused by factors such as infection, inflammation, or environmental stress. Tissue damage leading to necrosis can occur due to metabolic disturbances, failure to maintain cellular homeostasis, or damage to critical organelles such as mitochondria, the nucleus, and the cell membrane. In the context of infectious diseases, particularly TB, cell necrosis is a primary indicator of tissue damage caused by *M. tuberculosis* infection. The infection by *M. tuberculosis* triggers an inflammatory response that often leads to the formation of necrotic tissue in the lungs, commonly referred to as “caseous lesions” or “caseous necrosis.” This study is aimed at evaluating the level of epithelial cell necrosis in the lungs of mice infected with *M. tuberculosis* and assessing the potential of *Merremia mammosa* extract as a complementary therapy in repairing lung tissue damage caused by this infection.

*M. tuberculosis* infection in the lungs, both in mice and humans, often induces an intense immune response. Upon entering the body through inhalation of airborne *M. tuberculosis* bacteria, these bacteria target alveolar macrophages in the lungs. The bacteria are capable of surviving within the macrophages and avoiding destruction, leading to chronic inflammation. The immune system responds by forming granulomas composed of immune cells such as macrophages, T lymphocytes, and dendritic cells, which attempt to isolate the bacteria and prevent its spread. Simultaneously, excessive inflammatory responses can cause tissue damage, leading to necrosis.

Caseous necrosis is a distinctive form of necrosis observed in TB infections, where the necrotic tissue has a soft, cheese‐like texture. This damage occurs as a result of a combination of bacterial infection–induced cell death, excessive immune responses, and a lack of oxygen in the granuloma area, causing the cells within the affected tissue to die. At this stage, lung tissue damage becomes highly significant and can lead to permanent loss of lung function. This is one of the primary reasons why TB can cause long‐term damage and sustained pulmonary dysfunction in affected individuals [22].

### 3.2. Necrosis of Lung Cells in Mice Infected With *M. tuberculosis*

Necrosis of lung tissue due to *M. tuberculosis* infection typically occurs in the form of granulomas, which are characteristic structures formed as part of the immune response to infection. These granulomas consist of immune cells, such as macrophages, which attempt to contain the spread of bacteria but can also cause tissue damage. This damage may progress to necrosis if the infection persists or if the immune response is insufficient to control the infection. In TB, necrosis can occur in two main forms: cascade necrosis, where some cells die and damage surrounding tissue, and caseous necrosis, which occurs at the center of granulomas, where the tissue becomes hardened and cheese‐like in texture. This necrotic process leads to impaired lung function and contributes to clinical symptoms of TB, such as coughing up blood, shortness of breath, and weight loss. In this study, the number of epithelial cells undergoing necrosis was measured across five high‐power fields at 400× magnification for each mouse sample infected with *M. tuberculosis*. Evaluation was performed on groups of mice treated with *Merremia mammosa* extract and a control group that received no treatment. The data obtained show significant variability in the number of necrotic cells between the groups receiving *Merremia mammosa* extract treatment and those not treated. Table 1 presents the total number of necrotic cells and the average number of necrotic cells per field for each sample.

**Table 1:** Results of lung necrosis calculation using five high‐power fields at 400× magnification.

**Code**	**Total necrotic cell count**	**Average necrotic cells per high-power field**
P11	8	1.6
P12	2	0.4
P13	0	0.0
P14	4	0.8
P15	1	0.2
P16	2	0.4
P21	1	0.2
P22	2	0.4
P23	16	3.2
P24	3	0.6
P25	10	2.0
P26	1	0.2
P31	3	0.6
P32	1	0.2
P33	0	0.0
P34	1	0.2
P35	0	0.0
P36	0	0.0
P37	1	0.2
P41	1	0.2
P42	0	0.0
P43	1	0.2
P44	0	0.0
P45	2	0.4
P46	3	0.6
P47	0	0.0
K+1	1	0.2
K+2	0	0.0
K+3	0	0.0
K+4	1	0.2
K+5	0	0.0
K+6	8	1.6
K−1	0	0.0
K−2	—	—
K−3	0	0.0
K−4	8	1.6
K−5	1	0.2
K−6	0	0.0
K−7	6	1.2

*Note:* K− = control group, only given *Mycobacterium tuberculosis* bacteria; K+ = control/comparative group that was given *Mycobacterium tuberculosis* and rifampicin (P1, P2, and P3 each received a 2 mL/200 g body weight dose of extract orally).

#### 3.2.1. Variability in the Number of Necrotic Cells

In this study, the groups treated with *Merremia mammosa* extract, such as P11, P23, and P25, showed a higher number of necrotic cells compared to the negative control group (K−) and some positive control groups (K+). Group P23, which recorded 16 necrotic cells with an average of 3.2 necrotic cells per high‐power field, demonstrated significant tissue damage. This value is much higher than the negative control group (K−), which did not receive any treatment or infection and showed 0.0 necrotic cells in the observed field. This could be attributed to two main factors. First, a more severe *M. tuberculosis* infection in the P23 group may have led to a more intense immune response, eventually causing more severe tissue damage. *M. tuberculosis* infection can cause the formation of granulomas, which function to isolate the bacteria from healthy tissue. However, in some cases, excessive inflammation may lead to caseous necrosis, a type of necrosis that occurs in the granuloma area due to tissue damage caused by inflammatory cytokines and oxidative stress [23]. This condition often exacerbates lung tissue damage and impedes the healing process. In this study, higher doses of *Merremia mammosa* extract in some groups showed an impact on tissue damage levels. Although the compounds in *Merremia mammosa* extract have anti‐inflammatory and antibacterial properties, administering excessively high doses can affect the immune response balance, potentially worsening tissue damage [24].

However, some other treatment groups, such as P13, P33, P35, and P47, showed no necrotic cells, indicating that *Merremia mammosa* extract may help reduce cell necrosis. These results suggest that *Merremia mammosa* extract has the potential to exert a therapeutic effect in reducing tissue damage in some mice infected with *M. tuberculosis*. Despite the varying results, these findings point to the potential of *Merremia mammosa* extract in helping reduce cell necrosis in certain individuals. The ability of *Merremia mammosa* extract to reduce cell necrosis is likely related to its ability to attenuate excessive inflammatory responses, which often contribute to tissue damage.

*Merremia mammosa* extract is known to contain active compounds with anti‐inflammatory properties that can reduce the production of proinflammatory cytokines such as TNF‐*α* and IL‐1*β*. These cytokines play a crucial role in chronic inflammation and tissue damage occurring in TB [25]. By reducing inflammation, *Merremia mammosa* extract also has the potential to lower the high levels of oxidative stress, which is one of the causes of cell damage in *M. tuberculosis* infection. Reducing oxidative stress may help mitigate further damage to lung cells and support the healing process.

Thus, although higher doses of *Merremia mammosa* extract may increase tissue damage in some groups, in other groups, the extract demonstrated the ability to reduce cell necrosis and repair tissue damage. This suggests that the use of *Merremia mammosa* extract could be a promising alternative therapy for addressing lung tissue damage in *M. tuberculosis* infection. However, further research is needed to determine the optimal dosage and explore its mechanisms more deeply for this therapy to be effectively applied in TB treatment.

#### 3.2.2. Manja Roenigk Scoring and Necrotic Cell Count

The results of the study on the level of pulmonary cell necrosis in mice infected with *M. tuberculosis* show significant variation in the number of necrotic cells across the treatment groups. The assessment of cell necrosis in this study was performed using the Manja Roenigk scoring system, which provides an overview of the quality of cell damage based on a scale from 1 to 4. Using this scoring criterion, we can analyze the study results based on the average necrotic cell scores for each treatment group, including both the control and the *Merremia mammosa* extract‐treated groups.

From Table 1, a significant difference in the average number of necrotic cells between the groups treated with *Merremia mammosa* extract and the control groups can be observed. The following is a discussion of the study results, starting from Groups P11 to K−7, based on the data of necrotic cells counted per high‐power field and the Manja Roenigk scoring system. This discussion is aimed at delving deeper into the effects of *Merremia mammosa* extract treatment on cell necrosis in the lungs of *M. tuberculosis*–infected mice.

##### 3.2.2.1. P11 Group

• Total necrotic cells: 8
• Average necrotic cells per high‐power field: 1.6

**Figure 1:**
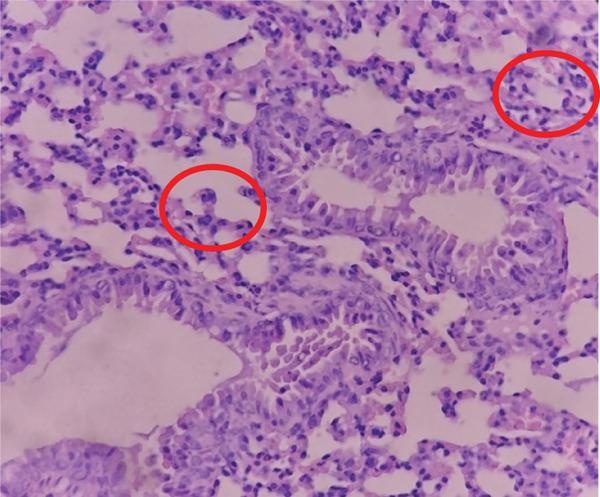
Histopathological image of lung tissue in Group P11. Red circles indicate necrotic cells showing nuclear condensation and loss of normal cellular structure.

The P11 group as shown in Figure 1 showed a total of eight necrotic cells, with an average of 1.6 necrotic cells per high‐power field. This value suggests that the P11 group tends to approach a score of 2, indicating the presence of parenchymal degeneration in the lung cells, although necrosis is not as extensive as in the other groups. This score reflects significant cell damage in some lung cells, but there are still some normal cells present. It is possible that the *Merremia mammosa* extract treatment in this group still has a therapeutic effect, although not fully.

##### 3.2.2.2. P12 Group

• Total necrotic cells: 2
• Average necrotic cells per high‐power field: 0.4

The P12 group showed a total of 2 necrotic cells, with an average of 0.4 necrotic cells per high‐power field. This value approaches a score of 1, which indicates that more cells are normal than those undergoing degeneration or necrosis. In this case, the treatment with *Merremia mammosa* extract demonstrates quite positive results in repairing lung tissue damage caused by *M. tuberculosis* infection. The P12 group can be considered the group that experienced significant tissue improvement, with minimal cell damage.

##### 3.2.2.3. P13 Group

• Total necrotic cells: 0
• Average necrotic cells per high‐power field: 0.0

The P13 group showed excellent results, with no necrotic cells detected at all. The average value of 0.0 indicates that the treatment with *Merremia mammosa* extract in this group was highly effective in reducing or even halting the process of lung cell necrosis. These results suggest that *Merremia mammosa* extract has significant potential in protecting lung cells from further damage. However, further research is needed to understand the exact mechanisms behind this protective effect.

##### 3.2.2.4. P14 Group

• Total necrotic cells: 4
• Average necrotic cells per high‐power field: 0.8

The P14 group showed a total of four necrotic cells, with an average of 0.8 necrotic cells per field of view. This number indicates that the lung cells experienced parenchymal degeneration, but the damage was not severe. This value is close to a score of 2, which suggests that the treatment with *Merremia mammosa* extract provides some protective effect on the lung tissue, although a portion of the cells still undergoes structural changes due to *M. tuberculosis* infection.

##### 3.2.2.5. P16 Group

• Total necrotic cells: 2
• Average necrotic cells per high‐power field: 0.4

The P16 group showed relatively good results, with two necrotic cells and an average of 0.4 necrotic cells per field of view. This value is close to a score of 1, indicating that treatment with *Merremia mammosa* extract in this group can reduce lung cell necrosis and help maintain the cells in a relatively healthy condition. Although there is some damage to a few cells, this treatment appears to have a positive impact in protecting lung tissue.

##### 3.2.2.6. P21 Group

• Total necrotic cells: 1
• Average necrotic cells per high‐power field: 0.2

The P21 group showed one necrotic cell with an average of 0.2 necrotic cells per field of view. This indicates that the majority of the lung cells remained normal and did not experience significant damage. Treatment with *Merremia mammosa* extract in this group appears to be quite effective in preventing lung tissue damage caused by *M. tuberculosis* infection, although there is a slight possibility that a few cells may have experienced mild damage.

##### 3.2.2.7. P22 Group

• Total necrotic cells: 2
• Average necrotic cells per high‐power field: 0.4

The P22 group had a total of two necrotic cells, with an average of 0.4 necrotic cells per field of view. Similar to other groups with comparable results, this group showed minimal damage to lung tissue, closer to a score of 1, indicating that treatment with *Merremia mammosa* extract was quite effective in reducing necrosis in lung cells.

##### 3.2.2.8. P23 Group

• Total necrotic cells: 16
• Average necrotic cells per high‐power field: 3.2

**Figure 2:**
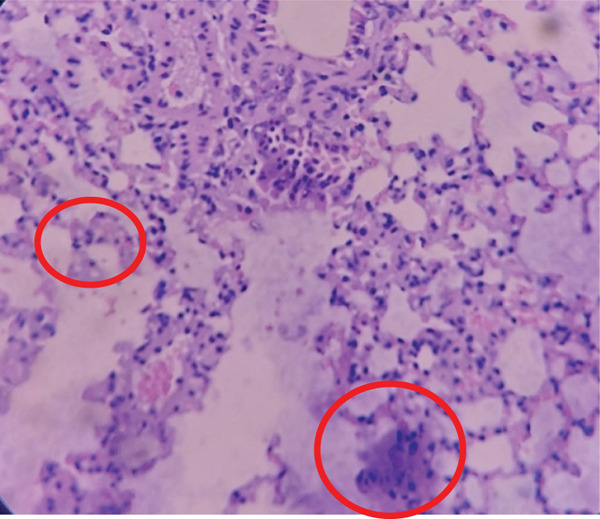
Histopathological image of lung tissue in Group P23. Red circles indicate necrotic cells in the lung tissue.

The P23 group as shown in Figure 2 showed striking results, with 16 necrotic cells and an average of 3.2 necrotic cells per field of view. This result is very close to a score of 4, indicating severe necrosis in the lung tissue. This could be due to a more severe *M. tuberculosis* infection or a higher dose of *Merremia mammosa* extract, which may have led to more intense inflammation in the lung tissue. The increase in necrosis score in this group warrants further attention, including a review of the dose or mechanisms that might be contributing to the elevated necrosis.

##### 3.2.2.9. P24–P47 Groups

**Figure 3:**
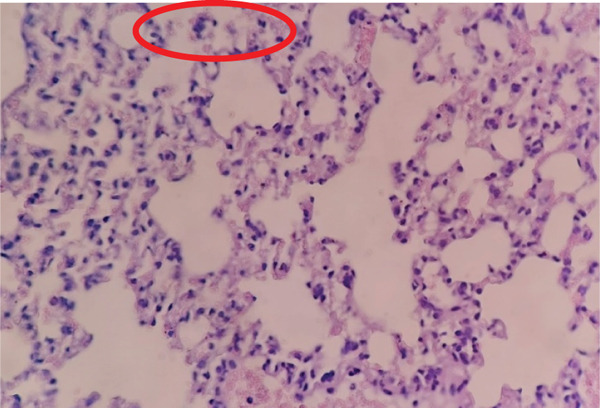
Histopathological image of lung tissue in Group P31. The red circle indicates necrotic cells in the lung tissue.

**Figure 4:**
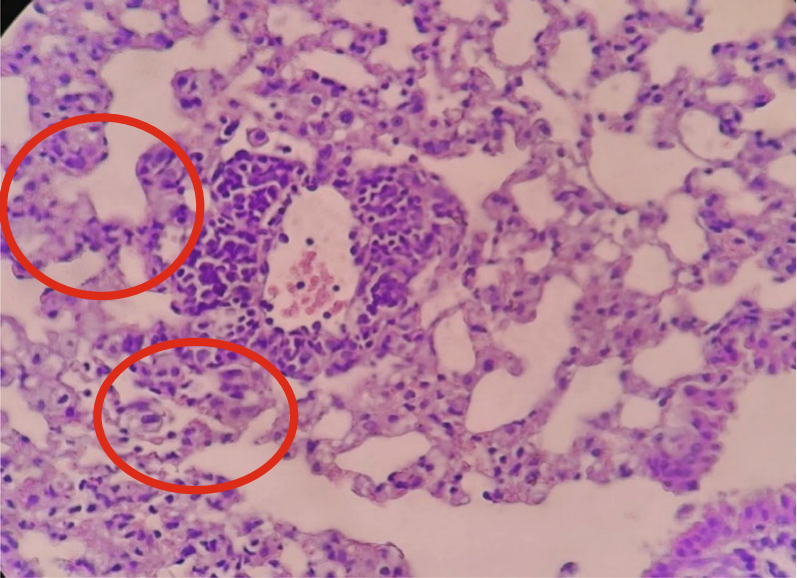
Histopathological image of lung tissue in Group P46. Red circles indicate necrotic cells in the lung tissue.

As shown in Figure 3, lung tissue from Group P31 exhibited mild inflammatory cell infiltration and limited necrotic foci, indicating moderate tissue response compared with other treatment groups. In general, the other groups exhibited a fairly consistent pattern, with some groups showing very few or no necrotic cells at all. Some groups, such as P24 (average 0.6), P25 (average 2.0), and P46 (average 0.6) as shown in Figure 4, showed signs of lung tissue damage, although the damage was not severe. This suggests that *Merremia mammosa* extract can offer some protection to lung tissue, but it does not completely eliminate the process of cell degeneration or necrosis.

##### 3.2.2.10. K+ and K− Groups

**Figure 5:**
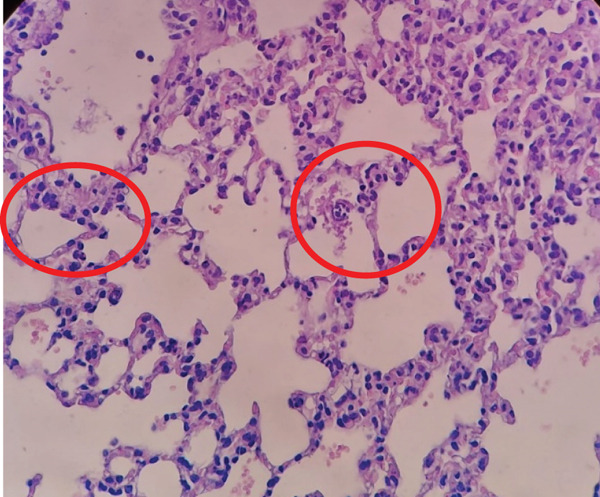
Histopathological image of lung tissue in Group K−7. Red circles indicate necrotic cells in the lung tissue.

**Figure 6:**
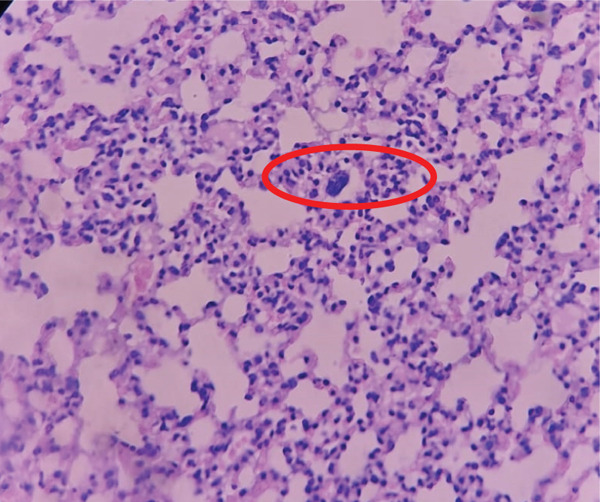
Histopathological image of lung tissue in Group K+1. The red circle indicates necrotic cells in the lung tissue.

The K+ and K− groups displayed greater variability. The positive control group (K+1, K+4, and K+6) and the negative control group (K−), as shown in Figures 5 and 6, showed varying average necrosis values. Overall, the negative control group (K−) exhibited the best results, with a necrosis score of zero (0.0). The positive control group, however, showed higher variability in necrosis, with some subgroups like K+4 and K−4 having higher average necrosis, though still not reaching severe levels of necrosis.

#### 3.2.3. Ekeftivitas Dosis Ekstrak *Merremia mammosa*

Most of the groups with lower or zero necrotic cell counts, such as P13, P33, and P47, may have received lower doses of *Merremia mammosa* extract. Lower doses may be more effective in preventing further damage to lung tissue without causing side effects or excessive immune responses. The significant reduction in cell necrosis in these groups suggests that proper dose regulation can lead to substantial improvement in tissue damaged by bacterial infection. However, groups like P23, which showed a high number of necrotic cells, may indicate that higher doses of *Merremia mammosa* extract could trigger an excessive inflammatory response. This highlights the importance of dose regulation in herbal therapy to ensure that optimal therapeutic effects are achieved without causing harmful side effects. The appropriate dosage of *Merremia mammosa* extract needs to be determined through further research to establish safe and effective tolerance limits for infected lung tissue [26].

The results of this study showed that administration of *Merremia mammosa* extract at 50 and 100 ppm reduced lung tissue necrosis compared to the control group, indicating its potential protective effect. However, at the highest concentration (150 ppm), necrosis scores were found to increase. This paradoxical finding may be associated with dose‐dependent toxicity, as has been reported in other herbal‐based therapies. Previous studies have shown that *Merremia mammosa* extract is generally safe at specific doses in animal models, but excessive concentrations may trigger cellular stress or hepatotoxic effects [4, 10]. Therefore, while *Merremia mammosa* demonstrates promise as an adjunct therapy against TB, careful dose optimization is required to balance efficacy and safety. Further toxicity studies, particularly at higher doses, are warranted to confirm these findings.

One of the factors to consider in this study is the individual response to *M. tuberculosis* infection. Each mouse may have a different susceptibility to this infection, which could influence the extent of tissue damage. In addition, factors such as age, general health status, and immune system function may also affect the outcomes of the study. Therefore, to obtain a more accurate picture of the effects of *Merremia mammosa* extract, it is important to consider individual variations in the analysis of the results.

The findings of this study are in line with previous research that showed plant extracts with anti‐inflammatory properties, such as *Merremia mammosa*, can help reduce tissue damage in *M. tuberculosis* infections. Research found that *Merremia mammosa* extract has potential in reducing inflammation in lung tissue, which, in turn, can reduce cell necrosis and enhance the healing process [27]. Active compounds in *Merremia mammosa* extract, such as flavonoids and saponins, are known to possess antioxidant activity that can reduce oxidative stress caused by the infection [28]. Furthermore, the antibacterial compounds in *Merremia mammosa* extract may help inhibit the growth of *M. tuberculosis*, thereby directly reducing infection and tissue damage.

*Merremia mammosa* is recognized in traditional medicine for its therapeutic benefits, including wound healing, anti‐inflammation, and infection treatment. This plant is found in many tropical areas and has been widely used in traditional healing. Modern studies have shown that *Merremia mammosa* contains active compounds with anti‐inflammatory, antibacterial, and antioxidant properties, which have potential in reducing tissue damage caused by infections, particularly in TB caused by *M. tuberculosis*.

Several mechanisms may explain how *Merremia mammosa* extract works: First, this extract has anti‐inflammatory potential that can reduce excessive inflammation. Chronic inflammation often causes damage to healthy tissue and exacerbates necrosis, especially in *M. tuberculosis* infections. The anti‐inflammatory compounds in *Merremia mammosa* work by inhibiting the production of proinflammatory cytokines such as TNF‐*α* and IL‐1*β*, which contribute to the inflammatory process and tissue damage.

Second, *Merremia mammosa* extract functions as an antioxidant. The inflammatory processes triggered by *M. tuberculosis* infection generate high oxidative stress, which damages healthy cells. The antioxidants in *Merremia mammosa* can reduce this oxidative damage, thus helping to protect lung cells from further damage and accelerate the healing process. Additionally, the antibacterial compounds in *Merremia mammosa* extract can help suppress the growth of *M. tuberculosis*. While not as potent as conventional drugs such as rifampicin in killing bacteria, this extract can help inhibit bacterial growth, thereby reducing infection and further tissue damage.

*Merremia mammosa* has been used in traditional medicine, particularly in Indonesia, to treat various health issues, including infectious diseases and inflammation. The roots, leaves, and stems of *Merremia mammosa* are often utilized to alleviate symptoms of respiratory diseases, such as coughs, colds, and even TB. With its anti‐inflammatory, antibacterial, and antioxidant properties, this plant can accelerate healing and repair lung tissue damaged by *M. tuberculosis* infection.

To evaluate the effectiveness of herbal treatments, scoring systems like the Manja Roenigk score are used to measure changes in infected tissue, particularly in cases of cell necrosis caused by bacterial infection. Based on this study, *Merremia mammosa* extract shows varied results in reducing cell necrosis in lung tissue. Some groups treated with *Merremia mammosa* extract showed a reduction in cell necrosis, while others experienced a greater increase in necrosis, likely due to higher doses or more severe infection conditions. Groups that did not show any cell necrosis, such as P13, P33, and P47, demonstrated better protection against lung tissue damage. This provides evidence that *Merremia mammosa* extract can help protect lung tissue from further damage, although the results vary depending on the dose and individual body conditions.

## 4. Conclusion

The level of cell necrosis in the lungs in this study showed significant variation among the treatment groups. Some groups that received *Merremia mammosa* extract, such as P23, exhibited a high level of necrosis, which may be attributed to either a more severe *M. tuberculosis* infection or a higher dose of the extract. On the other hand, other groups such as P13, P33, P35, and P47 showed zero necrosis, indicating that treatment with *Merremia mammosa* extract has the potential to reduce lung cell necrosis. The negative control group (K−) showed better results in terms of the number of necrotic cells, with some groups exhibiting no necrosis at all. This suggests that groups that either did not receive treatment or only received the infection experienced less tissue damage. Meanwhile, the positive control group (K+), which received rifampicin treatment, exhibited lower necrosis levels compared to some of the *Merremia mammosa* extract treatment groups, indicating that dose adjustment or changes in the extract type may be necessary to achieve optimal therapeutic effects.

The dose of *Merremia mammosa* extract affects the level of cell necrosis in the lungs. Groups with higher doses, such as P11 and P25, showed a significant increase in necrosis. Nevertheless, other factors such as the severity of the infection and the individual condition of the mice may also influence the results. Therefore, it is crucial to determine the optimal dose of *Merremia mammosa* extract that provides therapeutic effects without causing additional tissue damage. The use of the Manja Roenigk score proved effective in assessing lung tissue damage in mice infected with *M. tuberculosis*. This scoring system allows researchers to assess histological changes in lung tissue more objectively based on levels of cell necrosis, hydropic degeneration, and parenchymal degeneration. By using this score, researchers can more accurately evaluate the impact of the treatments on lung tissue and obtain more comprehensive data.

Overall, *Merremia mammosa* extract shows potential as a therapeutic agent in reducing cell necrosis in lungs infected with *M. tuberculosis*. Some treatment groups demonstrated the ability to improve lung tissue conditions and reduce the level of cell necrosis. However, the effectiveness of this extract in treating TB still requires further research, including the determination of the correct dosage and a deeper understanding of its mechanisms of action.

## Conflicts of Interest

The authors declare no conflicts of interest.

## Funding

This work was supported by Poltekkes Kemenkes Surabaya.

## Data Availability

The data supporting the findings of this article cannot be published due to privacy and confidentiality reasons.
